# Influence of cell type and cell culture media on the propagation of foot-and-mouth disease virus with regard to vaccine quality

**DOI:** 10.1186/s12985-018-0956-0

**Published:** 2018-03-16

**Authors:** Veronika Dill, Bernd Hoffmann, Aline Zimmer, Martin Beer, Michael Eschbaumer

**Affiliations:** 1 Institute of Diagnostic Virology, Friedrich-Loeffler-Institut, Südufer 10, 17493 Greifswald–Insel Riems, Germany; 2 Merck KGaA, Merck Life Sciences, Upstream R&D, Frankfurter Straße 250, 64293 Darmstadt, Germany

**Keywords:** Animal-component-free media, Foot-and-mouth disease virus, BHK21, Suspension cells, Serum-free media

## Abstract

**Background:**

Suspension culture of BHK cells allows large-scale virus propagation and cost-efficient vaccine production, while the shift to animal-component-free cell culture media without serum is beneficial for the quality and downstream processing of the product. Foot-and-mouth disease virus is still endemic in many parts of the world and high-quality vaccines are essential for the eradication of this highly contagious and economically devastating disease.

**Methods:**

Changes to the viral genome sequence during passaging in an adherent and a suspension cell culture system were compared and the impact of amino acid substitutions on receptor tropism, antigenicity and particle stability was examined. Virus production in suspension cells in animal-component-free media and in serum-containing media as well as in adherent cells in serum-containing media was compared. Infection kinetics were determined and the yield of intact viral particles was estimated in all systems using sucrose density gradient centrifugation.

**Results:**

Capsid protein sequence alterations were serotype-specific, but varied between cell lines. But The A_24_-2P virus variant had expanded its receptor tropism, but virus neutralization tests found no changes in the antigenic profile in comparison to the original viruses. There were no differences in viral titer between a suspension and an adherent cell culture system, independent of the type of media used. Also, the usage of a serum-free suspension culture system promoted viral growth and allowed an earlier harvest. For serotype O isolates, no differences were seen in the yield of 146S particles. Serotype A preparations revealed a decreased yield of 146S particles in suspension cells independent of the culture media.

**Conclusion:**

The selective pressure of the available surface receptors in different cell culture systems may be responsible for alterations in the capsid coding sequence of culture-grown virus. Important vaccine potency characteristics such as viral titer and the neutralization profile were unaffected, but the 146S particle yield differed for one of the tested serotypes.

**Electronic supplementary material:**

The online version of this article (10.1186/s12985-018-0956-0) contains supplementary material, which is available to authorized users.

## Background

Foot-and-mouth disease virus (FMDV) is a highly transmissible and extremely contagious RNA virus, infecting domestic as well as wild cloven-hooved animals [1]. Vaccination campaigns are the way of choice to eradicate FMDV in endemic countries and in case of an outbreak in an FMDV-free country, vaccination is a useful strategy to limit spread [2].

Vaccines produced to combat FMDV have a long history, going back to first attempts in the early 1900s and Waldmann’s first inactivated vaccine, developed in 1937 [3]. The most common production cell line is the mammalian baby hamster kidney cell (BHK21, clone 13), adapted to grow in suspension by Capstick et al. [4], and processed for large-scale fermenters by Telling and Elsworth [5]. To achieve adaption of the virus to the production cell line, the virus is first passaged in BHK21 adherent cells until a rapid cytopathogenic effect develops, and then further propagated and expanded in stationary or roller systems [6]. In industry practice, viruses are passaged in suspension cell culture to expand the virus to large scale. Preparation of master and working seed stocks for the vaccine production process are the last steps of a successful virus adaption in both cell culture systems [7]. In the vaccine production process, the raw materials, including serum-containing cell culture media, need special attention. Serum as well as other components such as animal tissue hydrolysates are poorly defined, resulting in significant lot-to-lot variation of the product [8, 9]. On top of their substantial costs, animal-derived products can contain viruses, mycoplasmal bacteria or prions, and therefore require special risk assessments by the supplier and the user [9, 10]. Because of this, attempts to find alternatives to serum in vaccine production have been of major importance for many years [11].

Today, cell culture media can be divided in different types based on their content of animal-derived products. Serum-free media (SFM) do not require the addition of serum for optimal cell growth but may contain other additives derived from animals such as lactalbumin, casein, insulin, lipids or sterols [12]. Animal-component-free media (ACFM) are media in which none of the components are animal-derived [11]. Protein-free media (PFM) are free of supplemental polypeptide factors but may contain hydrolyzed peptide fragments from animal or plant sources. Finally, chemically defined media (CDM) comprise well-characterized constituents of low molecular weight and are, in most cases, free of proteins [11, 12]. BHK21 cells have already been adapted to grow in serum-free or animal-component-free cell media for rabies vaccine production [13, 14]. With adaption to serum-free conditions, BHK21 cells switch from anchorage-dependent to suspension growth [13, 14] and fundamental changes in cell structure take place [15, 16]. On the other hand, selective pressures during the adaption of viral strains to BHK21 cells, whether as adherent or as suspension cells, can lead to capsid alterations that influence the antigenicity and stability of the virus particle.

The first part of the study examines the adaption of the virus to an adherent and a suspension cell culture system, the viral sequence changes that take place during subsequent passaging as and their possible impact on receptor tropism, particle stability and antigenicity. The second part of the study compares virus production in an animal-component-free medium and virus production in serum-supplemented growth medium and the possible differences in quality and quantity of the viral harvest.

## Methods

### Cells

The adherent BHK21C13 cell line (CCLV-RIE 179 in the Collection of Cell Lines in Veterinary Medicine, Friedrich-Loeffler-Institut [FLI], Greifswald, Germany; originally derived from the American Type Culture Collection (ATCC) specimen CCL-10™; short: BHK179) and the adherent BHK21 “clone Tübingen” cell line (CCLV-RIE 164, short: BHK164) were cultured in Minimum Essential Medium Eagle (MEM), supplemented with Hanks’ and Earle’s salts (Sigma, St. Louis, USA) with 10% fetal bovine serum (FBS) during maintenance and passaging, and with 5% FBS during infection experiments. Cells were incubated in flasks with sealed caps at 37 °C.

The suspension cell line BHK21C13-2P (originally derived from the European Collection of Authenticated Cell Cultures specimen 84,111,301; short: BHK-2P) was either maintained in Glasgow MEM (Thermo Fisher Scientific), supplemented with tryptose phosphate (Sigma-Aldrich) and sodium hydrogen carbonate (Carl Roth GmbH + Co. KG, Karlsruhe, Germany) with 5% FBS or was adapted to grow in the animal-component-free medium Cellvento™ BHK-200 (Merck KGaA, Darmstadt, Germany) in TubeSpin® bioreactors (TPP Techno Plastic Products AG, Trasadingen, Switzerland). The cells were maintained in a shaker incubator with 320 rpm (rpm) at 37 °C, 5% CO_2_ and 80% relative humidity.

The Chinese hamster ovary (CHO) cell lines CHO-K1 (ATCC CCL-61, held as CCLV-RIE 134), lacking the known FMDV integrin receptors [17], and the heparan sulfate (HS)-deficient CHO677 [18] (CRL 2244, held as CCLV-RIE 1524) were maintained in Ham’s MEM mixed 1:2 with Iscove’s Modified Dulbecco’s Medium (Thermo Fisher Scientific) and with 10% FBS at 37 °C in sealed flasks.

### Viruses and virus titrations

The FMDV isolates A_24_ Cruzeiro and O_1_ Manisa were selected from archival stocks at the FLI. Their passage history and origin can be found in Additional file 1: Table S1.

Viral titers were estimated by endpoint titration with the Spearman-Kärber method [19, 20] and expressed as 50% tissue culture infectious dose (TCID_50_) per milliliter. Titrations for virus grown on all cell lines were performed on the adherent BHK164 to avoid biasing the results by titrating BHK179-passaged virus on BHK179 cells.

### Virus adaption and passaging

Both virus strains were serially passaged on BHK179 monolayers for 20 passages. In suspension cells, the viruses were passaged until stable adaption to the suspension cell line was achieved. Adaption was defined as a decrease in cell viability to values under 10% within less than 24 h post infection (hpi). Adaption of the virus to growth in BHK-2P as well as passaging the virus on BHK179 was done two times independently. FMDV strain A_24_ Cruzeiro was fully adapted to BHK-2P after 19 passages (16 in the second experiment) and O_1_ Manisa after 22 (19) passages. The adapted viruses will be referred to as A_24_–179, A_24_-2P, O_1_–179 and O_1_-2P, respectively.

### RNA extraction, RT-PCR and sequencing

FMDV RNA of the original stocks of A_24_ Cruzeiro and O_1_ Manisa, of the virus passage 20 in BHK179 and of the final passages in BHK-2P of both adaption experiments was extracted using TRIzol® LS Reagent (Invitrogen, Carlsbad, CA, USA) and the RNeasy® Mini Kit (Qiagen, Valencia, CA, USA) according to the manufacturers’ instructions. A previously described method was used for RT-PCR and sequencing of the nearly complete open reading frame [21].

The nucleotide sequences were assembled and mapped with Geneious (Biomatters Limited) against the complete published sequence for A_24_ Cruzeiro (GenBank accession no. AY593768) and O_1_ Manisa (AY593823) followed by an alignment of original and passaged virus sequences.

To find the passage in which each mutation was fixed in the suspension system, the passages in which a rapid drop in cell viability was observed for the first time were chosen for additional sequencing. For the adherent cell system no such indicator existed and therefore the passages were sequenced in arbitrary intervals.

### Structure analysis

Amino acid sequences of the original virus and the final passages in BHK179 and BHK-2P were used to model virus capsid protomers using the Geno3D algorithm [22]. The X-ray crystal structures of A_24_ Cruzeiro [23] (Protein Data Bank accession 1ZBE) and O_1_ Manisa [24] (1FOD) served as templates. In total, ten possible structures were generated and the best-fitting model was further analyzed with the UCSF Chimera package [25]. Chimera was developed by the Resource for Biocomputing, Visualization, and Informatics at the University of California, San Francisco (supported by NIGMS P41-GM103311). VIPERdb [26] was used to extract contact information for specific residues.

### *In-silico* analysis

The complete genomes of FMDV strains representing possible vaccine strains [27] as well as representative strains for different topotypes within the seven serotypes were downloaded from GenBank. Multiple sequence alignments for all serotypes were performed using the MUSCLE algorithm as implemented in Geneious and the amino acids at the positions of interest were tabulated.

### Acid sensitivity

The protocol of Martín-Acebes et al. [28] was used with modifications. Equal amounts of virus (A_24_ Cruzeiro and O_1_ Manisa, original isolates as well as adapted to BHK179 and BHK-2P) were mixed at a final dilution of 1:100 with phosphate-buffered saline (PBS) solutions of different pH within the range of pH values commonly seen in the suspension cell system (7.5, 7.0, 6.8, 6.5). An additional solution with a pH of 5.5 was used as a positive control for FMDV inactivation. The mixtures were incubated for 30 min at room temperature and then neutralized with 1 M Tris-HCl (pH 8.0). The remaining infectivity in each sample was determined by titration on BHK164 cells as described above. Experiments were performed three times independently.

### Infectivity testing on CHO cells

A procedure described by Jackson et al. [29] was used to quantify the capacity of the virus strains to infect the FMDV receptor-deficient cell lines CHO-K1 and CHO677. As a modification of the original protocol, the CHO cell preparations were titrated on BHK164. The test was conducted in duplicates and performed three times independently.

### Virus neutralization test (VNT)

The VNT was performed on BHK164 cells with A_24_ Cruzeiro, A_24_–179, A_24_-2P, O_1_ Manisa, O_1_–179, and O_1_-2P as described by the World Organization for Animal Health (OIE) [30]. A bovine serum, collected 21 days after infection with an earlier passage of the A_24_Cruzeiro stock virus, was used to neutralize the serotype A virus isolates. Another bovine serum, from an animal infected with an earlier passage of the O_1_ Manisa stock virus, also collected at 21 dpi, was used to examine the serotype O virus isolates. The highest dilution in which 50% of the wells did not show any CPE defined the neutralization titer. Titers are expressed as the log_10_ of the reciprocal of that dilution. To determine the relationship between the original and adapted virus isolates, the r_1_ value was calculated by dividing the neutralization titer against the adapted isolate by the neutralization titer against the original virus isolate [30]. All experiments were performed independently in duplicates for a total of three times.

### Virus infection kinetics

BHK-2P cells were seeded at a density of 1 × 10^6^ cells/mL and infected with the adapted A_24_-2P or O_1_-2P at an MOI of 0.1. BHK179 cells were cultured in T25 culture flasks until confluency and infected with A_24_–179 or O_1_–179 under the same conditions as the BHK-2P cells. Samples to determine the viral titer were taken after 0 and 4 h and then every 2 h until a total incubation time of 24 h.

Because the determination of cell death and viability is different between adherent and suspension cells, cytopathic effect (CPE, in %) was documented for BHK179 cells, while cell number and cell viability were assessed for BHK-2P cells. Cell death in suspension cell culture cannot be visually evaluated under a microscope and therefore determination of cell viability is necessary. Additionally, the cell density of an infected culture is compared to an equally seeded negative culture to account for the rapid growth of a healthy suspension culture. Cell numbers and cell viability have been determined by trypan blue staining with an automated cell counter (TC20™, Bio-Rad).

### Determination of viral yield

Adherent and suspension cells (cell count 3.7 × 10^7^) were infected at a multiplicity of infection (MOI) of 0.1 and incubated for 20 h. The supernatant was clarified of cell debris by centrifugation for 10 min at 3200×g at 4 °C, followed by purification through a 30% (wt/vol) sucrose cushion in 40 mM sodium phosphate buffer (pH 7.6) with 100 mM NaCl (buffer P as in [31]), centrifuged at 125,755×g in a SW32Ti rotor (Beckman Coulter, Optima LE-70) for 2 h 50 min at 10 °C. Pellets were resuspended in 400 μL buffer P and loaded onto 15% to 45% (wt/vol) sucrose gradients in buffer P. Ultracentrifugation was performed in a SW32Ti rotor at 96,281×g for 3 h at 10 °C. Gradients were fractionated from the bottom of the gradient into one milliliter fractions. All fractions were heated to 70 °C for 30 min before analysis. Absorption at 260 nm was measured twice with a spectrophotometer (NanoDrop™ 2000, Thermo Fisher Scientific). FMDV protein was detected in duplicate by a standard serotype-specific double-antibody sandwich ELISA [30]. The experiment was performed three times.

### Statistical analysis and data presentation

Linear mixed-effects models using R (http://www.r-project.org) and lme4 [32] were used to evaluate the differences between treatment groups, with replicates as random effects. The packages car and phia were applied to calculate Wald chi-square tests for fixed effects and their interactions. *P*-values < 0.001 were taken as significant.

## Results

### Viral sequence changes differ between adaption to adherent or suspension cells for A_24_ Cruzeiro, but not for O_1_ Manisa

Viral sequence changes in the capsid coding region were examined during passaging for at least 15 passages in a conventional adherent culture system with serum and in a suspension culture system using animal-component-free media. The O_1_ Manisa virus acquired three heterologous mutations within the capsid coding region. All substitutions occurred in VP1 (K41 N, E83K and K210E) and were the same in both culture systems. The first mutation is the substitution of a positively charged lysine at position 41 with a polar uncharged asparagine. This amino acid is flanked by a non-polar phenylalanine at position 39 and a positively charged arginine. It is located at an interface between two VP1 molecules in the five-fold symmetry. Residue E83 of VP1 is exposed on the outer capsid surface in the DE-loop with no interactions at any interfaces. Thirdly, at position 210, the positively charged lysine shifted to a negatively charged glutamic acid. In both culture systems these mutations were already acquired during the first passages (see Additional file 1: Table S1.1 and S1.2). In addition, *in-silico* analyses at the respective positions revealed a degree of conservation for the original amino acids of 97 to 100% (see Additional file 2: Table S2).

A_24_ Cruzeiro also developed amino acid differences in the capsid coding region between low and high passage virus. A_24_–179 acquired heterologous mutations in VP1 (E194K) and VP3 (C56R). Originally, a negatively charged glutamic acid was situated at position 194; by passage 20, it had been replaced by a positively charged lysine. The *in-silico* analysis revealed a degree of conservation of 93% towards a negatively charged amino acid at this particular position, 7% of examined viruses exhibited a hydrophobic amino acid but none had a positive charge (see Additional file 2: Table S2). On position 56 of VP3, an uncharged cysteine was replaced with a positively charged arginine, another change that contributes to a net increase of positive charges on the surface of the virus capsid. This amino acid change could be found in only 3% of all examined serotype A sequences in the database (see Additional file 2: Table S2). In general, the mutations in A_24_–179 do not seem to be strictly fixed in the genome and occurred lately in the course of passaging (see Additional file 1: Table S1.3).

**Fig. 1:**
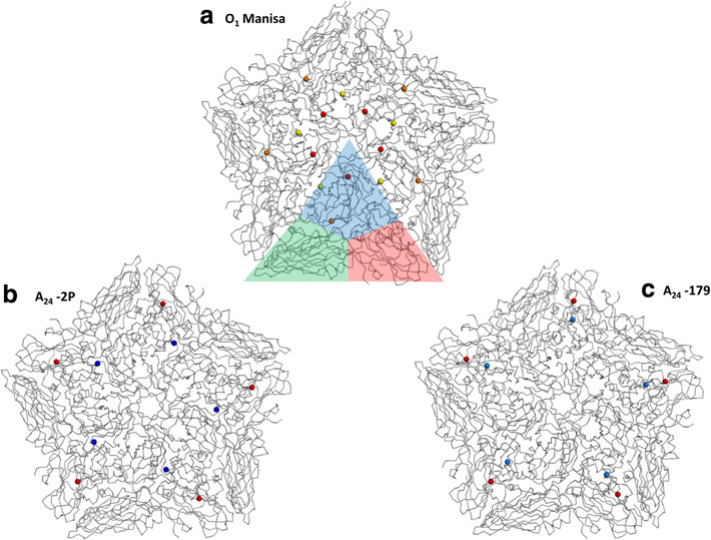
3D structure with mutations acquired during adaption of FMDV strains A_24_ Cruzeiro and O_1_ Manisa. Panels (**a**), (**b**), and (**c**) present detailed structural models of the virus capsid pentamers. The X-ray crystal structure of 1FOD served as template for the 3D models. The structure of a single protomer is highlighted in panel (**a**) (blue: VP1, green: VP2, red: VP3). While O_1_ Manisa (panel **a**) acquired the same three mutations in the VP1 region of the capsid (K210E: yellow dots, E83K: orange dots, K41 N: red dots) independent of the culture system, A_24_ Cruzeiro developed different mutations in VP1 (blue dots) and VP3 (red dots) in adherent cell culture (panel **c**) and suspension cell culture (panel **b**). However, these mutations lie in similar locations on the particle

In A_24_-2P, the changes were also located in VP1 and VP3, but at different positions (VP1: E95K, VP3:H85Q). In VP1, a shift from a negatively charged glutamic acid to a positively charged lysine occurred at position 95. This amino acid is in close proximity to the uncharged valine 29 and tyrosine 30 of VP3 and constitutes the interface between two VP1 molecules in the five-fold axis of the molecule. Similar to the mutation in VP1 of A_24_–179, a negatively charged amino acid at this position is conserved among 73% of serotype A isolates, while 27% display a hydrophobic amino acid. No examined isolate had a positively charged amino acid (see Additional file 2: Table S2). The mutation in VP3 is located in a 3_10_ helix that forms the base of the depression of the HS-binding pocket. Here, a positively charged histidine was replaced by an uncharged glutamine on position 85, which strongly contradicts the conservation of a positively charged amino acid at this position of 100% (see Additional file 2: Table S2). Both mutations were acquired right from the start of the adaption process (see Additional file 1: Table S1.4). The results of this section are summarized in Additional file 3: Table S3. In addition, Fig. 1 illustrates the individual substitutions mapped to a capsid pentamer. For serotype O in particular, the mutations were distributed in a crown-like pattern on the surface in the center of the pentamer (Additional file 4: Figure S1).

### Mutations that allow binding to the HS binding pocket lead to increased acid sensitivity

**Fig. 2:**
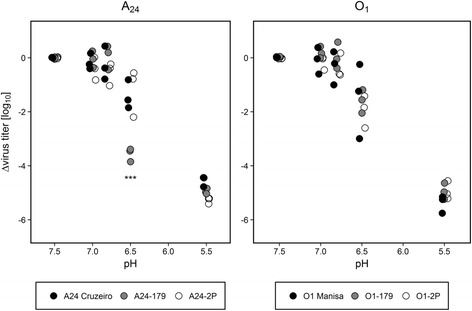
Sensitivity of O_1_ Manisa and A_24_ Cruzeiro and their derivatives to acidic pH Equal amounts of virus of the different passages and culture systems were incubated in buffers of different pH for 30 min, then neutralized and titrated. Titers are expressed relative to the value obtained using PBS at pH 7.5. Experiments were performed three times independently. Significance code: “***” *p* ≤ 0.001

To test if any of the acquired mutations affect the integrity of the viral particle, its stability under acidic conditions was examined. Incubation of the original viruses and their derivatives in buffer solutions of different pH revealed no significant differences in acid stability for the different O_1_ Manisa virus populations. Down to a pH of 6.8, no decrease in titer was visible for any of the virus isolates. Variability in virus titer increased at pH 6.5 but the virus populations did not differ significantly from each other. The positive control at pH 5.5 led to a drastic decrease in virus titer. The viral titer for the different A_24_ virus population remained stable down to pH 6.8 as well. However, at pH 6.5, A_24_–179 underwent a significantly stronger decrease in virus titer than the original virus isolate and A_24_-2P (Fig. 2).

### Capsid mutations extend receptor usage but do not negatively affect neutralization profiles

To examine if the observed sequence changes enable the passaged viruses to use non-integrin cellular receptors, infection experiments with FMDV receptor-deficient cell lines were performed. CHO-K1 cells, which express HS but no relevant integrin receptors, were infected with the original isolates as well as passaged viruses. All six viruses were able to grow on CHO-K1 cells. No significant difference was found between O_1_–179 and O_1_-2P, but both isolates grew to significantly lower titers in CHO-K1 cells than the original O_1_ Manisa (*p* < 0.001). A_24_-2P infected CHO-K1 cells significantly more effectively than A_24_ Cruzeiro, A_24_–179 and all O_1_ isolates. Furthermore, it was the only virus isolate that was able to infect CHO677 cells, which are devoid of both surface integrins and HS (Table 1).

**Table 1:** Growth on receptor-deficient cells and neutralization profiles of A_24_ Cruzeiro and O_1_ Manisa and their derivatives

Virus isolate	Cell line		VNT	
	CHO-K1	CHO677	Serum titer	r_1_-value
A_24_ Cruzeiro	2.8 ± 0.2	negative	3.4 ± 0.2	
A_24_–179	2.3 ± 0.3	negative	3.6 ± 0.2	1.05
A_24_-2P	4.0 ± 0.4	2.5 ± 0.6	3.5 ± 0.1	1.02
O_1_ Manisa	2.8 ± 0.1	negative	3.2 ± 0.1	
O_1_–179	2.0 ± 0.1	negative	3.5 ± 0.2	1.09
O_1_-2P	1.9 ± 0.2	negative	3.4 ± 0.1	1.06

Values represent mean virus and neutralization titers and standard deviations, shown as log_10_ infectious or neutralizing doses per milliliter. The r_1_-value was calculated by dividing the neutralization titer against the adapted isolate by the neutralization titer against the original virus isolate

To determine if any antigenic changes took place during adaption, virus neutralization tests were performed with bovine sera raised against the original A_24_ and O_1_ viruses. Titers for A_24_–179 and A_24_-2P were similar to the titer obtained with A_24_ Cruzeiro, with r_1_-values ≥1.0. The same was true for O_1_ Manisa and its derivatives. (Table 1).

### The viral yield is similar in adherent and suspension cell culture but the peak is reached earlier in suspension cells.

Virus infection kinetics were performed to compare the pace of viral growth and the overall yield, estimated by endpoint titration, in adherent and suspension BHK cells in serum-containing growth medium and animal-component-free-growth medium. The progression of cell death together with the viral titer was documented to find the best time point for virus harvest in the different cell media systems.

**Fig. 3:**
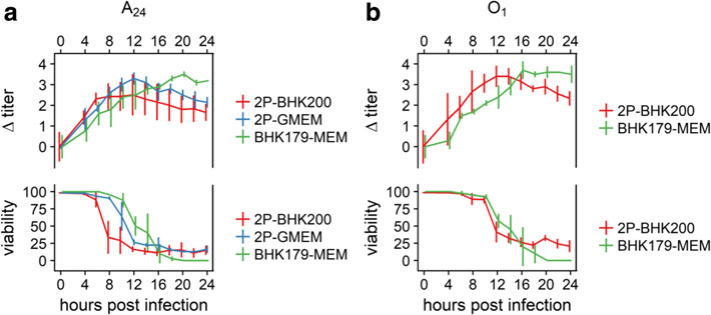
Virus infection kinetics on monolayer and suspension BHK cells. Virus infection kinetics were performed using the virus variants A_24_-2P to infect the suspension BHK-2P cell line, maintained in BHK200 (red line) or GMEM + 5% FCS (blue line) and A_24_–179 to infect the monolayer BHK179 cell line (green line) at an MOI = 0.1 (panel **a**). The virus variant O_1_-2P was used to infect the suspension BHK-2P cell line, maintained in BHK200 (red line), and the virus variant O_1_–179 was used to infect the monolayer BHK179 cell line (green line) under the same conditions (panel **b**) as described for serotype A. All preparations were sampled for the first time after 4 h and every 2 h thereafter. CPE and cell viability are given in percent (%). Titers are shown in log_10_ TCID_50_ relative to time 0

The highest viral titers occur in the moment the cell viability drops drastically in the suspension cell culture system or when the cytopathic effect (CPE) reached 100% in adherent BHK cells. The comparison of both cell culture types shows an earlier cell viability drop and virus release in the suspension cell line for both serotypes compared to the adherent BHK cells. Additionally, the virus infection in the suspension cell system proceeds even faster when using ACFM compared to serum-containing growth medium. After its peak, all tested conditions showed a slight decrease in viral titer over time, independent of cell culture media, cell culture system or serotype used. For serotype A, the maximum titers were reached after 8 h in the suspension cell system with ACFM and after 12 h in the suspension cell system with serum-containing growth medium. The peak of virus titer in adherent BHK cells was seen after 20 h. The progression of serotype O was similar: maximum titers for O_1_-2P developed after 12 h in suspension cell culture and after 16 h for O_1_–179 in adherent BHK cells. No significant differences in the viral yield were found between the tested conditions (Fig. 3).

The maximum titers that were reached were 8.8 ± 0.4 log_10_ TCID_50_ per mL for O_1_–179 and 8.6 ± 0.1 for A_24_–179. On BHK-2P, the viral titers were similar between serotypes and independent of the culture medium: 8.3 ± 0.5 log_10_ TCID_50_/mL for O_1_-2P in ACFM, 7.6 ± 0.6 for A_24_-2P in ACFM and 7.7 ± 0.3 for A_24_-2P in serum-containing medium.

### Evaluation of viral particle integrity

Preparations of A_24_–179 and O_1_–179 in a serum-containing adherent cell system, A_24_-2P in both an ACFM and a serum-containing suspension cell system as well as O_1_-2P in an ACFM suspension cell system were analyzed on sucrose density gradients to compare the yield of intact 146S particles, empty 75S capsids and free RNA. The presence of nucleic acid in a fraction was quantified by measuring its absorbance at 260 nm with a spectrophotometer. In the antigen ELISA, the presence of FMDV proteins is indicated by an increased absorbance at 492 nm. Empty particles (75S), which do not contain nucleic acid, do not absorb at 260 nm, but can be detected with the antigen ELISA.

**Fig. 4:**
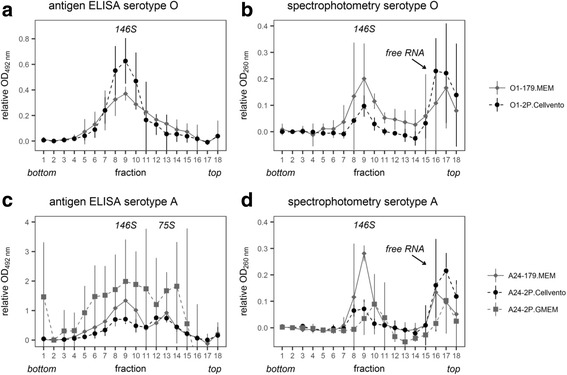
Sucrose gradient profiles of passaged A_24_ Cruzeiro and O_1_ Manisa. Identical numbers of BHK179 and BHK-2P cells, maintained in GMEM + 5% FCS or ACFM were infected with an MOI = 0.1 and virus was harvested after 20 hpi. The harvested virus was concentrated by ultracentrifugation and sedimented through a 15–45% sucrose density gradient. The peaks corresponding to 146S (fraction 9) and empty 75S particles (fraction 13) are indicated. FMDV protein content is shown as absorbance at 492 nm obtained with antibodies specific for O_1_ (panel **a**, − 179: solid, grey line, -2P: dotted, black line) and A_24_ (panel **c**, − 179: solid, grey line, -2P: dotted, black line for ACFM; dotted, grey line for serum-containing GMEM) in a standard FMDV antigen ELISA. RNA content corresponds to absorbance at 260 nm as measured with a spectrophotometer (panel **b**: O_1_ isolates, panel **d**: A_24_isolates, − 179: solid, grey line, -2P: dotted, black line for ACFM; dotted, grey line for serum-containing GMEM). For both assays, absorbances were normalized by subtracting the absorbance reading of fraction 2

The O_1_-2P isolate showed higher amounts of intact 146S virus particles in the ELISA (peak at fraction 9) in comparison to O_1_–179, but spectrophotometric measurements revealed a lower 146S peak and high amounts of free RNA for O_1_-2P. None of these differences were statistically significant (Fig. 4, panel a, b).

For A_24_ Cruzeiro, the A_24_–179 isolate had significantly higher amounts of 146S particles (fraction 9) in the spectrophotometric measurements than the A_24_-2P preparations, independent of the cell culture medium used (Fig. 4, panel d). No significant differences between A_24_–179 and A_24_-2P grown in ACFM were found in the antigen ELISA. Although the A_24_-2P isolate grown in suspension cells with serum-containing media has a higher peak in fraction 9, these data were collected with a different batch of ELISA plates and cannot be directly compared to the earlier preparations.. Formation of empty virus particles (75S, peak at fraction 13) was evident for all serotype A virus preparations (Fig. 4, panel c). For A_24_–179, the amount of empty particles was reduced compared to the amount of 146S particles, but for both A_24_-2P preparations the amount of 75S particles was approximately equal to the amount of intact 146S particles.

## Discussion

BHK21 cells have been shown to change during passaging and even lose their susceptibility for FMDV [33]. Additionally, the change from adherent to suspension cell culture comes along with profound changes to the cells that the virus needs to adapt to [34]. The first part of this study focused on the sequence changes in the viral genome that take place when adapting to either an adherent or a suspension BHK cell culture system and their influence on virus receptor tropism, antigenicity and particle stability.

The passaging of FMDV O_1_ Manisa in either cell culture system resulted in three substitutions in the VP1 capsid protein (K41 N, E83K, K210E). Earlier studies by Gullberg and colleagues found that the switch from K to E at position 210 at the VP1/2A junction in a serotype O FMDV results in the formation of virus particles containing the uncleaved VP1-2A product [31] and is also linked to the E83K substitution within VP1 [35].This substitution is responsible for the inhibition of the cleavage of the VP1/2A junction [35] and provides a selective advantage in the BHK cell culture system, but did not enable the virus to successfully infect CHO cells [36, 37].

The third substitution K41 N is located close to the fivefold symmetry axis of the virus particle at the interface between two VP1 molecules and results in a reduction of positive charge at the interface similar to K210E. Mutations in this particular region have been implicated in the ability to infect cells independently of receptors such as integrin, HS, chondroitin sulfate or sialic acid [38–40]. While it is not clear what selective advantage the inhibition of the VP1/2A junction might have, in sum these mutations seem to allow the use of a receptor on BHK21 cells that is neither integrin nor HS. However, experiments with receptor-deficient CHO cell lines revealed no extended tropism of O_1_-2P and O_1_–179 in comparison to the original O_1_ Manisa isolate.

For A_24_ Cruzeiro, the acquired substitutions E194K in VP1 and C56R in VP3 in A_24_–179 reflect an adaption for utilization of HS as receptor. According to Fry et al., the HS binding pocket consists of three sites: VP3 residues 55–60 form one of the walls, while residues 84–88 shape the base. The other two walls are composed of residue 133–138 of VP2 and the C-terminus of VP1 (residues 195–197) [23]. The amino acid change from histidine to arginine at position 56 of VP3 has been described as a characteristic feature for HS attachment of serotype O viruses [41]. Together with the second substitution (E194K in VP1), the HS-binding pocket of the capsid acquires a clearly more positive charge. While type A FMDV might show lower affinity to HS than type O [42], the beta-B “knob” regions between residues 55 to 62 of VP3 are structurally very similar between the serotypes [23]. A previous study examining mutations in serotype A capsid proteins after cell culture adaptation also found a switch of C56 to R in a BHK21 culture system, while the E194K mutation was only fixed in strains passaged on IB-RS-2 cells and in a small minority of BHK21 derived isolates [40].

The sequence changes in A_24_-2P were not as distinct. The switch from a positively charged amino acid to a neutral glutamine (H85Q) at the base of the HS-binding pocket does not support the acquisition of HS as receptor during the course of cell culture adaption. Similar to O_1_ Manisa, the A_24_-2P isolate obtained a positively charged amino acid (E95K) close to the fivefold symmetry axis in VP1. As already discussed for O_1_ Manisa, these substitutions suggest that A_24_-2P uses a yet unknown “third” receptor on BHK-2P suspension cells. This assumption is supported through studies using an A_24_ Cruzeiro mutant (A-SIR #42) that harbors the same E95K change in VP1 [17]. Further studies revealed this amino acid change to be responsible for utilizing Jumonji C-domain containing protein 6 to infect cells in an integrin- and HS-independent way [43]. Indeed, in the present study, A_24_-2P was the only mutant capable of infecting CHO677 cells, which do neither have surface integrins nor HS.

It has already been shown that adherent BHK cells offer a limited range of surface molecules such as integrins that can be utilized as receptors by FMDV, and BHK cells in suspension culture often have none at all [34]. For this reason, there is a selective pressure in favor of alternative entry mechanisms for the virus. The observed differences between the serotypes may indicate that FMDV type A is more malleable or has a higher mutation rate, resulting in a more variable adaption to different BHK cell lines. Conversely, FMDV serotype O may have a preference for certain mutations that result in a more universal outcome of adaption. This hypothesis is supported by a recently published study by Anil and colleagues, which also showed different mutations occurring in an FMDV serotype A strain depending on passaging in suspension or monolayer BHK cells [44].

Nevertheless, important vaccine quality aspects such as viral antigenicity and particle stability appear to be unaffected by the acquired amino acid substitutions. The r_1_-value, which determines the serological relationship between the original virus and the passaged mutant [30], was higher than 1 for all isolates, indicating that the neutralizing epitopes on the capsid surface are unchanged and immunization with the passaged viruses confers protection like the original isolate does. As for particle stability, one of the main reasons for instability is an increased sensitivity towards low pH. Viral genome release inside the cell is induced through endosomal acidification [28] and there are known sequence mutations that lead to a more labile or a more stable virus capsid. For the O_1_ Manisa viruses in the study, no differences in acid sensitivity were observed. In contrast, the virus variant A_24_–179 showed a significantly stronger decrease in viral titer at pH 6.5 than the original virus or A_24_-2P. Several studies indicate that virion stability is influenced through amino acid replacements preferentially located at the N terminus of VP1 or the pentameric interface [27, 45, 46]. However, none of the previously described mutations or amino acid substitutions were detected in any of the virus variants generated during passaging in this study.

The switch from media that contain serum and other animal-derived components to a serum-free or even completely animal-component-free system is a major step forward in the production of vaccines. It can bring many advantages such as lower cost, reduced risk of contamination and a cleaner product recovery [9, 12]. The second part of the study examined virus production in an animal-component-free medium compared to virus production in serum-supplemented growth medium and their influence on quality and quantity of the viral harvest.

In the production process of an FMDV vaccine, the virus is harvested as soon as the majority of the cells are dead [8]. Therefore, viral infection kinetics were recorded to find the best time point to harvest the virus. In a monolayer cell culture system, the total cell count is limited due to the available surface area, but growth in a suspension cell system is rapid and unlimited as long as sufficient nutrients and oxygen are available [47]. The maximum viral titers of the kinetic experiments were similar between both cell culture systems. These results are consistent with other studies comparing roller and suspension systems [45]. Furthermore, the maximum viral titers were not influenced by the type of cell culture medium. Kinetic experiments even revealed a quicker virus release when using ACFM. This might be because ACFM contain fewer inhibitory ingredients [46]. On top of reduced biological risks and lower costs through the use of ACFM, the shorter process time also could be an important factors to be taken into account for vaccine producers. The decrease in post-peak titers over time was independent of serum content in the media.

Sucrose density gradient profiles revealed no significant differences in the content of 146S particles between BHK-2P in ACFM and BHK179 in serum-containing media for serotype O preparations. For serotype A preparations, there were no significant differences in contents of 146S particles between the preparation in ACFM and serum-containing growth medium, but the viral yield from the adherent cell culture system exhibited a significantly higher content of 146S particles than the suspension cell preparations. It is also striking that A_24_-2P yielded 146S and 75S particles in nearly equal amounts, independent of the cell culture media. In addition, all preparations contained high amounts of free RNA. This might lead to the assumption that the packaging of viral RNA into the particle is impaired, which leads to increased free RNA and empty capsid formation. However, not all of the free RNA is of viral origin. The process of purifying the virus from the cell culture supernatant does not completely remove free cellular RNA, which then accumulates in the top fraction of the sucrose gradient.

## Conclusion

This study found serotype-specific capsid alterations dependent on the cell line or clone the virus was adapted to. However, cell-specific adaption did not change the neutralization profile of the passaged viruses compared to the original isolates. No differences were found in viral growth and titer between the different cell and media systems. The use of ACFM even appears to support faster virus replication. Differences in the yield of 146S particles, however, were dependent on the cell line, rather than influenced by the culture medium.

## Additional files

Additional file 1: **Table S1.1.** Nucleotide changes in the VP1 coding region of FMDV type O_1_ Manisa during serial passaging in adherent BHK21 cells. **Table S1.2.** Nucleotide changes in the VP1 coding region of FMDV type O_1_ Manisa during serial passaging in BHK-2P suspension cells. **Table S1.3.** Nucleotide changes in the capsid-coding region of FMDV type A_24_ Cruzeiro during serial passaging in adherent BHK21 cells. **Table S1.4.** Nucleotide changes in the capsid-coding region of FMDV type A_24_ Cruzeiro during serial passaging in BHK-2P suspension cells. (DOCX 17 kb)

Additional file 2: **Table S2.** *In-silico* analysis covering possible additional vaccine strains (A) and most topotypes within the different serotypes (B) of FMDV. (DOCX 35 kb)

Additional file 3: **Table S3.** Summary of virus isolates, cell culture systems and mutations acquired during passaging. (DOCX 13 kb)

Additional file 4: **Figure S1.** Side view of pentamer 3D structure with mutations acquired during adaption of FMDV strains A_24_ Cruzeiro and O_1_ Manisa. Panel A shows the crown-like distribution of the acquired mutations in the VP1 region of the capsid of O_1_ Manisa (K210E: yellow dots, E83K: orange dots, K41 N: red dots). The substituted amino acids in O_1_ Manisa are clustered around the symmetry axis of the pentamer and are more prominent on the capsid surface than the mutations in A_24_-2P (Panel B) and A_24_–179 (Panel C) (VP1: blue dots, VP3: red dots). (PPTX 788 kb)

## Abbreviations

ACFM: Animal-component-free media
ATCC: American Type Culture Collection
BHK: Baby hamster kidney
CCLV-RIE: Collection of Cell Lines in Veterinary Medicine, Riems
CDM: Chemically defined media
CHO: Chinese hamster ovary
CPE: Cytopathic effect
FBS: Fetal bovine serum
FLI: Friedrich-Loeffler-Institut
FMDV: Foot-and-mouth disease virus
Hpi: Hours post infection
HS: Heparan sulfate
MEM: Minimum Essential Medium Eagle
MOI: Multiplicity of infection
OIE: World Organisation for Animal Health
PBS: Phosphate-buffered saline
PFM: Protein-free media
RNA: Ribonucleic acid
rpm: Revolutions per minute
RT-PCR: Reverse transcription-polymerase chain reaction
SFM: Serum-free media
TCID_50_: 50% tissue culture infectious dose
VNT: Virus neutralization test
wt/vol: Weight/volume
