# Supplementary material for: Influence of cell type and cell culture media on the propagation of foot-and-mouth disease virus with regard to vaccine quality
Source: Virol J. 2018 Mar 16;15:46. doi: 10.1186/s12985-018-0956-0 (PMC5857075; doi:10.1186/s12985-018-0956-0)
Supplement: Supplementary file 1 — Table S1.1. Nucleotide changes in the VP1 coding region of FMDV type O1 Manisa during serial passaging in adherent BHK21 cells. Table S1.2. Nucleotide changes in the VP1 coding region of FMDV type O1 Manisa during serial passaging in BHK-2P suspension cells. Table S1.3. Nucleotide changes in the capsid-coding region of FMDV type A24 Cruzeiro during serial passaging in adherent BHK21 cells. Table S1.4. Nucleotide changes in the capsid-coding region of FMDV type A24 Cruzeiro during serial passaging in BHK-2P suspension cells. (DOCX 17 kb) [file 12985_2018_956_MOESM1_ESM.docx]

| **Table S2:** *In-silico* analysis covering possible additional vaccine strains (A) and most topotypes within the different serotypes (B) of FMDV. | | | | | | | | | | |
| --- | --- | --- | --- | --- | --- | --- | --- | --- | --- | --- |
|  |  |  |  |  |  |  |  |  |  |  |
| **S2.A** |  | **O_1_-179 / -2P** | | |  | **A_24_-179** | | **A_24_-2P** | |  |
|  |  | **K210E** | **E83K** | **K41N** |  | **E194K / VP1** | **C56R / VP3** | **E95K / VP1** | **H85Q / VP3** |  |
| **serotype O** | AF506822 | K | E | K |  | H | H | E | H | Foot-and-mouth disease virus O strain China/1/99(Tibet), complete genome |
|  | AY593818 | K | E | K |  | H | R | E | H | Foot-and-mouth disease virus O isolate o1campos iso96, complete genome |
|  | AY593819 | K | E | K |  | H | H | E | H | Foot-and-mouth disease virus O isolate o1campos94 iso94, complete genome |
|  | AY593823 | K | E | K |  | H | H | E | H | Foot-and-mouth disease virus O isolate o1manisa iso87, complete genome |
|  | FJ175666 | K | E | K |  | H | H | E | H | Foot-and-mouth disease virus - type O isolate Israel 07-6387, complete genome |
|  | GU566044 | K | E | K |  | H | - | E | H | Foot-and-mouth disease virus - type O isolate SUD/4/99 VP1 (1D) gene, partial cds |
|  | GU566058 | K | E | K |  | H | - | E | H | Foot-and-mouth disease virus - type O isolate SUD/3/2005 VP1 (1D) gene, partial cds |
|  | GU566063 | K | E | K |  | H | - | E | H | Foot-and-mouth disease virus - type O isolate SUD/8/2008 VP1 (1D) gene, partial cds |
|  | HQ009509 | K | E | K |  | H | H | E | H | Foot-and-mouth disease virus - type O strain China/5/99(Fujian), complete genome |
|  | JX040500 | K | E | K |  | H | H | E | H | Foot-and-mouth disease virus - type O isolate TUR/27/2011, complete genome |
|  | KR265075 | K | E | K |  | H | H | E | H | Foot-and-mouth disease virus - type O isolate O/UKG, complete genome |
| **serotype A** | KU208000 | K | S | K |  | E | C | E | H | Foot-and-mouth disease virus - type A isolate IND 40/2000 capsid protein gene, partial cds |
|  | KX002202 | K | D | K |  | E | C | V | H | Foot-and-mouth disease virus - type A isolate A/973_SL_Pede polyprotein gene, partial cds |
|  | KX002204 | K | D | K |  | E | C | V | H | Foot-and-mouth disease virus - type A isolate A/AGLopez01 polyprotein gene, partial cds |
|  | KX002205 | K | D | K |  | E | C | V | H | Foot-and-mouth disease virus - type A isolate A/ATLauquen01 polyprotein gene, partial cds |
|  | KY825717 | K | D | K |  | E | C | E | H | Foot-and-mouth disease virus - type A isolate A22/IRQ/24/64 polyprotein gene, partial cds |
|  | KF112902 | K | E | K |  | A | C | E | H | Foot-and-mouth disease virus - type A isolate A/EGY/1/2006 capsid protein gene, partial cds |
|  | FJ755037 | K | D | K |  | E | - | V | H | Foot-and-mouth disease virus - type A isolate A/IRN/27/2005 VP1 (1D) gene, partial cds |
|  | FJ755042 | K | D | K |  | E | - | V | H | Foot-and-mouth disease virus - type A isolate A/IRN/33/2005 VP1 (1D) gene, partial cds |
|  | AY593768 | K | E | K |  | E | C | E | H | Foot-and-mouth disease virus A isolate a24cruzeiro iso71, complete genome |
| **serotype Asia-1** | AY304994 | K | T | K |  | D | R | K | H | Foot-and-mouth disease virus Asia 1 IND 63/72, complete genome |
|  | JF739177 | K | T | K |  | D | R | K | H | Foot-and-mouth disease virus - type Asia 1 isolate As1/Shamir/89, complete genome |

| **serotype C** | AY593806 | K | T | K |  | Q | T | V | H | Foot-and-mouth disease virus C3 isolate c3ind iso19, complete genome |
| --- | --- | --- | --- | --- | --- | --- | --- | --- | --- | --- |
| **SAT serotypes** | AY593845 | K | V | L |  | - | D | V | N | Foot-and-mouth disease virus SAT 1 isolate sat1bot iso47, complete genome |
|  | KJ999940 | - | G | H |  | - | N | P | H | Foot-and-mouth disease virus - type SAT 2 isolate SAT2/BOT/18/98 P1 polyprotein gene, partial cds |
|  | AY593851 | K | A | H |  | Y | D | H | H | Foot-and-mouth disease virus SAT 3 isolate sat3-3bech iso29, complete genome |
|  | AY593853 | K | L | H |  | Y | D | P | H | Foot-and-mouth disease virus SAT 3 isolate sat3-4bech iso23, complete genome |

| **S2.B** |  | **O_1_-179 /O_1_-2P** | | |  | **A_24_-179** | | **A_24_-2P** | |
| --- | --- | --- | --- | --- | --- | --- | --- | --- | --- |
|  |  | **K210E** | **E83K** | **K41N** |  | **E194K / VP1** | **C56R / VP3** | **E95K / VP1** | **H85Q / VP3** |
| **Serotype A** | AY593751 | K | D | K |  | E | C | E | H |
|  | AY593753 | K | D | K |  | E | C | E | H |
|  | AY593757 | K | E | K |  | E | C | E | H |
|  | AY593760 | K | D | K |  | E | C | E | H |
|  | AY593761 | K | E | K |  | E | R | E | H |
|  | AY593771 | K | D | K |  | E | C | E | H |
|  | AY593777 | K | D | K |  | E | C | E | H |
|  | AY593778 | R | D | K |  | E | C | E | H |
|  | AY593780 | R | D | K |  | E | C | E | H |
|  | AY593785 | K | D | K |  | E | C | V | H |
|  | AY593789 | K | N | K |  | E | C | E | H |
|  | AY593793 | K | D | K |  | E | C | E | H |
|  | AY593794 | K | D | K |  | E | C | V | H |
|  | EF117837 | K | D | K |  | E | C | V | H |
|  | HM854025 | K | S | K |  | E | C | E | H |
|  | JF749843 | K | E | K |  | A | C | E | H |
|  | KC440881 | K | Q | K |  | E | C | E | H |
|  | KC440882 | K | E | K |  | E | C | E | H |
|  | KJ754939 | K | E | K |  | E | C | E | H |
|  | KP940474 | K | Q | K |  | E | C | E | H |
|  | KT968663 | K | T | Q |  | E | C | E | H |
|  |  |  |  |  |  |  |  |  |  |
| **Serotype O** | AF506822 | K | E | K |  | H | H | E | H |
|  | AJ539140 | K | E | K |  | H | H | E | H |
|  | AY593823 | K | E | K |  | H | H | E | H |
|  | AY593826 | K | E | K |  | H | H | E | H |
|  | EF175732 | K | E | K |  | Q | H | E | H |
|  | FJ175666 | K | E | K |  | H | H | E | H |
|  | GU384683 | K | E | K |  | H | H | E | H |
|  | HM229661 | K | E | K |  | H | H | E | H |
|  | JX040500 | K | E | K |  | H | H | E | H |
|  | JX869188 | K | E | K |  | H | H | E | H |
|  | KC440883 | K | E | K |  | H | H | E | H |
|  | KJ825809 | K | K | K |  | H | H | E | H |
|  | KR265075 | K | E | K |  | H | H | E | H |
|  | KU821591 | K | E | K |  | H | H | E | H |
|  | LC036265 | K | E | K |  | H | H | E | H |
|  |  |  |  |  |  |  |  |  |  |
| **Serotype Asia-1** | AY593796 | K | T | K |  | D | R | K | H |
|  | AY593797 | K | T | K |  | D | R | K | H |
|  | AY593800 | K | T | K |  | D | R | K | H |
|  | DQ989309 | K | T | K |  | D | R | K | H |
|  | DQ989321 | K | T | K |  | D | R | K | H |
|  | DQ989323 | K | T | K |  | D | R | K | H |
|  | EF614458 | K | T | K |  | D | R | K | H |
|  | GU125645 | K | T | K |  | D | R | K | H |
|  | GU125646 | K | T | K |  | D | R | K | H |
|  | HQ113233 | K | T | K |  | D | R | K | H |
|  | HQ631363 | K | T | K |  | D | R | K | H |
|  | HQ632774 | K | T | K |  | D | R | K | H |
|  | JN006719 | K | T | R |  | D | R | K | H |
|  | JN006720 | K | T | R |  | D | R | K | H |
|  | KU360085 | K | T | K |  | D | R | K | H |
|  |  |  |  |  |  |  |  |  |  |
| **C** | AF274010 | K | T | E |  | Q | M | V | H |
|  | KM268897 | K | T | K |  | Q | T | V | H |
|  |  |  |  |  |  |  |  |  |  |
| **Serotype SAT 1** | AY593839 | K | T | L |  | H | D | E | N |
|  | AY593840 | K | T | K |  | H | D | V | N |
|  | AY593841 | K | T | L |  | H | D | V | N |
|  | AY593842 | K | T | L |  | H | D | V | T |
|  | AY593844 | K | T | L |  | H | D | V | N |
|  | AY593846 | K | T | L |  | H | D | E | N |
|  | HM067706 | K | N | L |  | H | D | L | N |
|  | JF749860 | K | T | L |  | H | D | E | N |
|  | KM268899 | K | T | L |  | H | D | E | N |
|  | KU821590 | K | T | L |  | H | D | V | N |
|  |  |  |  |  |  |  |  |  |  |
| **Serotype SAT 2** | AF540910 | K | E | H |  | Y | N | P | H |
|  | AY593847 | E | E | H |  | Y | N | P | H |
|  | AY593848 | E | T | H |  | Y | N | P | H |
|  | AY593849 | E | E | H |  | Y | D | P | H |
|  | HM067704 | E | N | H |  | Y | N | P | H |
|  | HM067705 | E | E | H |  | Y | N | P | H |
|  | JF749861 | E | A | H |  | Y | N | P | H |
|  | JF749862 | E | K | H |  | Y | N | P | H |
|  | JF749864 | E | E | H |  | Y | N | P | H |
|  | JX014255 | E | D | H |  | Y | N | P | H |
|  | JX014256 | E | D | H |  | Y | N | P | H |
|  | KC440884 | E | D | H |  | Y | N | P | H |
|  | KM268900 | E | D | H |  | Y | N | P | H |
|  |  |  |  |  |  |  |  |  |  |
| **SAT3** | AY593850 | K | S | H |  | Y | D | H | C |
|  | AY593851 | K | N | H |  | Y | D | H | S |
|  | AY593852 | K | N | H |  | Y | D | H | S |
|  | AY593853 | K | T | H |  | Y | D | H | S |
|  | KJ820999 | K | D | H |  | Y | D | E | T |
|  | KM268901 | K | T | H |  | Y | D | H | S |
|  | KR108950 | K | T | H |  | Y | D | H | S |
|  | KX375417 | K | T | H |  | Y | D | H | S |

| colour legend: |  |  |  |
| --- | --- | --- | --- |
|  |  |  |  |
| amino acids with electrically **negative** charged side chains: | | | **D, E** |
| amino acids with electrically **positive** charged side chains: | | | **R, H, K** |
| amino acids with **polar uncharged** side chains: | | | **S, T, N, Q** |
| amino acids with **hydrophobic** side chains: | | | **A, V, I, L, M, F, Y, W** |
| special cases: |  |  | **C, U, G, P** |
