# Supplementary material for: Influence of cell type and cell culture media on the propagation of foot-and-mouth disease virus with regard to vaccine quality
Source: Virol J. 2018 Mar 16;15:46. doi: 10.1186/s12985-018-0956-0 (PMC5857075; doi:10.1186/s12985-018-0956-0)
Supplement: Supplementary file 3 — Table S3. Summary of virus isolates, cell culture systems and mutations acquired during passaging. (DOCX 13 kb) [file 12985_2018_956_MOESM3_ESM.docx]

**Table S3:** Summary of virus isolates, cell culture systems and mutations acquired during passaging.

| **virus** | **cell culture system** | **substitution** | **capsid protein** | **degree of conservation*** | **final passage**  **(1st/2nd experiment)** |
| --- | --- | --- | --- | --- | --- |
| A_24_-179 | adherent | E194K | VP1 | 93% | 20/20 |
|  |  | C56R | VP3 | 97% |  |
| A_24_-2P | suspension | E95K | VP1 | 73% | 19/16 |
|  |  | H85Q | VP3 | 100% |  |
| O_1_-179 | adherent | K41N | VP1 | 100% | 20/20 |
|  |  | E83K | VP1 | 96% |  |
|  |  | K210E | VP1 | 100% |  |
| O_1_-2P | suspension | K41N | VP1 | 100% | 22/19 |
|  |  | E83K | VP1 | 96% |  |
|  |  | K210E | VP1 | 100% |  |

*Degree of conservation is based on the results of the *in-silico* analyses at the respective positions for the particular serotypes (Table S2).
