# Supplementary figures and images for: Influence of cell type and cell culture media on the propagation of foot-and-mouth disease virus with regard to vaccine quality
Source: Virol J. 2018 Mar 16;15:46. doi: 10.1186/s12985-018-0956-0 (PMC5857075; doi:10.1186/s12985-018-0956-0)

## Slide 1
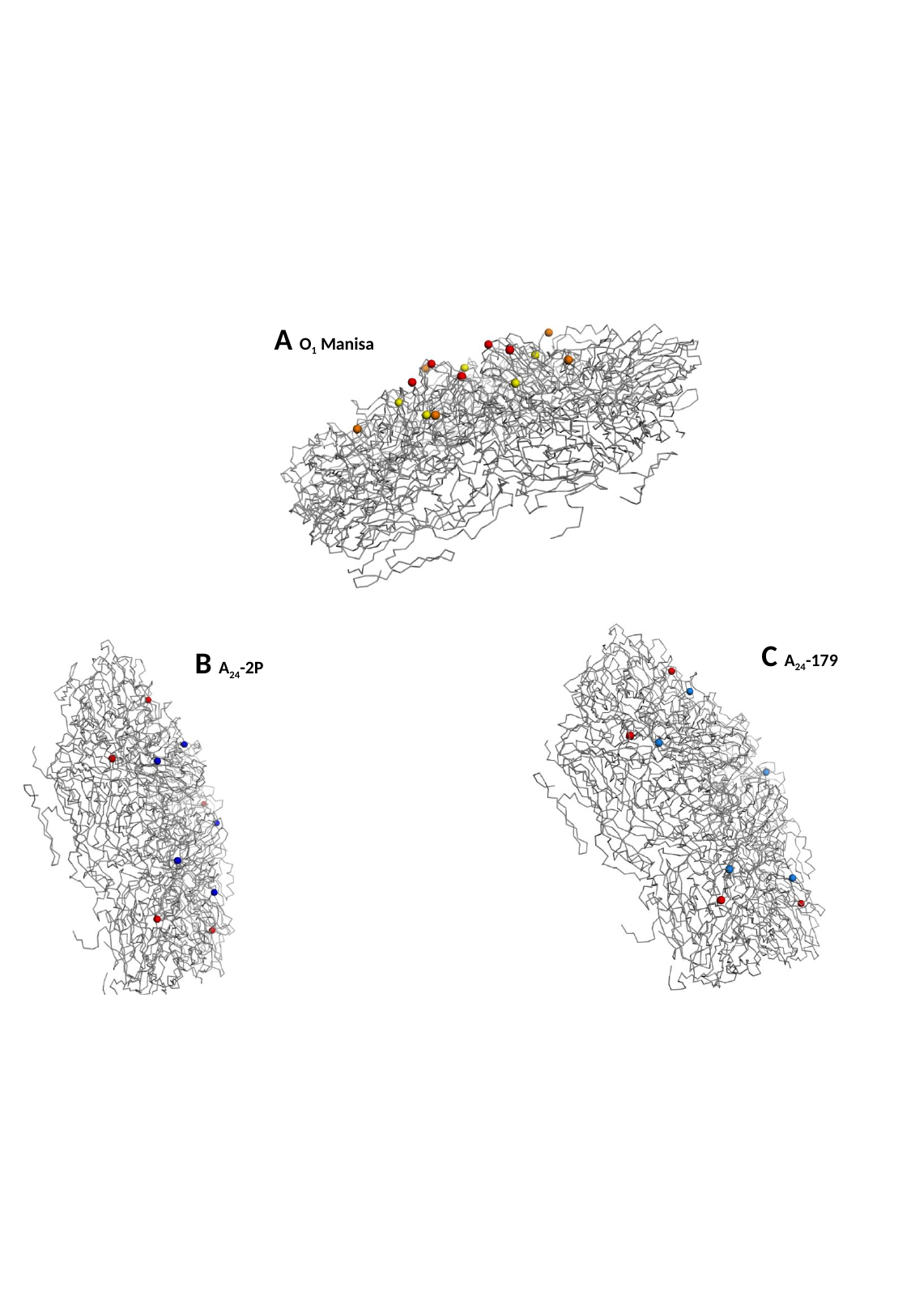

A O1 Manisa
C A24-179
B A24-2P

Supplement: Supplementary file 4 — Figure S1. Side view of pentamer 3D structure with mutations acquired during adaption of FMDV strains A24 Cruzeiro and O1 Manisa. Panel A shows the crown-like distribution of the acquired mutations in the VP1 region of the capsid of O1 Manisa (K210E: yellow dots, E83K: orange dots, K41 N: red dots). The substituted amino acids in O1 Manisa are clustered around the symmetry axis of the pentamer and are more prominent on the capsid surface than the mutations in A24-2P (Panel B) and A24–179 (Panel C) (VP1: blue dots, VP3: red dots). (PPTX 788 kb) [file 12985_2018_956_MOESM4_ESM.pptx]
